# Mesenchymal Stem Cells and Extracellular Vesicles in Osteosarcoma Pathogenesis and Therapy

**DOI:** 10.3390/ijms222011035

**Published:** 2021-10-13

**Authors:** Virinder Kaur Sarhadi, Ravindra Daddali, Riitta Seppänen-Kaijansinkko

**Affiliations:** Department of Oral and Maxillofacial Diseases, University of Helsinki and Helsinki University Hospital, 00014 Helsinki, Finland; ravindra.daddali@oulu.fi (R.D.); riitta.seppanen-kaijansinkko@helsinki.fi (R.S.-K.)

**Keywords:** osteosarcoma, mesenchymal stem cells, extracellular vesicles, therapy

## Abstract

Osteosarcoma (OS) is an aggressive bone tumor that mainly affects children and adolescents. OS has a strong tendency to relapse and metastasize, resulting in poor prognosis and survival. The high heterogeneity and genetic complexity of OS make it challenging to identify new therapeutic targets. Mesenchymal stem cells (MSCs) are multipotent stem cells that can differentiate into adipocytes, osteoblasts, or chondroblasts. OS is thought to originate at some stage in the differentiation process of MSC to pre-osteoblast or from osteoblast precursors. MSCs contribute to OS progression by interacting with tumor cells via paracrine signaling and affect tumor cell proliferation, invasion, angiogenesis, immune response, and metastasis. Extracellular vesicles (EVs), secreted by OS cells and MSCs in the tumor microenvironment, are crucial mediators of intercellular communication, driving OS progression by transferring miRNAs/RNA and proteins to other cells. MSC-derived EVs have both pro-tumor and anti-tumor effects on OS progression. MSC-EVs can be also engineered to deliver anti-tumor cargo to the tumor site, which offers potential applications in MSC-EV-based OS treatment. In this review, we highlight the role of MSCs in OS, with a focus on EV-mediated communication between OS cells and MSCs and their role in OS pathogenesis and therapy.

## 1. Introduction

Osteosarcoma (OS) is the most predominant primary bone cancer, commonly occurring in the long bones of children and adolescents [1]. OS is highly malignant and the major complications in OS arise due to a lack of immune response, leading to irregular bone growth and distant metastases, seen commonly in the lungs and liver. The current treatment approaches for OS are preoperative chemotherapy, surgical resection, and postoperative chemotherapy, which are effective in patients with localized OS. Conversely, patients with advanced, metastatic, and recurrent OS develop resistance to chemotherapy, which makes it difficult to treat, resulting in a poor prognosis [2]. Despite multidisciplinary treatments, there has been no change in the prognosis during the past two decades. The overall 5-year survival rate of OS patients is 65% in the case of localized disease, while it is 20% in those with metastasis, and significantly lower in those with lung metastasis [3]. The high heterogeneity and genetic complexity of OS make it challenging to identify new therapeutic targets [4].

A thorough understanding of the tumor microenvironment (TME), especially the bone microenvironment (BME), cellular crosstalk, and the molecular mechanisms underlying tumor progression, is essential for drug design and for developing new drug molecules for OS treatment. The BME is composed of the extracellular matrix (ECM) and a variety of cells, which includes mesenchymal stem cells (MSCs), endothelial cells, macrophages, stem cells, fibroblasts, osteoblasts, osteoclasts, and osteocytes that are organized to maintain the bone rigidity and the structural as well as functional integrity of the bone niche. All these cells together play a crucial role in normal bone development and bone physiology and can also lead to osteosarcoma in aberrant conditions.

MSCs are multipotent, non-hematopoietic cells that have the potential to self-rejuvenate and to differentiate into different cell types, including muscle cells, hepatocytes, osteoblasts, adipocytes, chondrocytes, and stromal cells [5,6]. MSCs interact with cancer and other cells in the tumor microenvironment via paracrine factors and through extracellular vesicles (EVs) to support tumor growth, progression, and metastasis. Contrary to their tumor-supportive role, they have also been implicated in tumor suppression. Hence, it is vital to understand the interaction of MSCs and OS cells in the tumor microenvironment to develop new and more effective OS therapy, and to overcome drug resistance. In this review, we discuss the role of MSCs in OS and highlight the reciprocal interaction of MSCs and OS cells in modulating each other’s functions, with a focus on the role of extracellular vesicles in MSC–OS communication and their therapeutic applications.

## 2. Mesenchymal Stem Cells’ Role in Osteosarcoma

MSCs are an important source of adult stem cells, present in many tissues, especially in bone marrow, and adipose tissue, and are involved in tissue repair and healing. MSCs play a significant role in OS pathogenesis, from the possible cells of origin of OS to its supportive role in OS growth, progression, metastasis, and drug resistance. OS is assumed to originate at some stage in the differentiation process of MSC to pre-osteoblast and the stage of differentiation of MSC affects the OS phenotype [7,8].

### 2.1. Cellular Origin of OS

A better understanding of the cellular origin of OS is essential to improve the outcomes of patients. There are controversial reports about the cellular origins of OS. Some propose that OS develops from MSC; by contrast, others suggest osteoblast precursor cells as the cells of origin of OS [9].

MSCs can differentiate into osteoblasts, chondroblasts, or fibroblasts, and OS can have an osteoblastic, chondroblastic, or fibroblastic phenotype, based on the predominant cancer cell type, which suggests that MSC might be the cells of origin of OS [10,11]. There is evidence suggesting that OS originates at some point during the differentiation of MSC to pre-osteoblast [7,8]. The argument that MSC is a cell of origin of OS is supported by studies in murine models, where MSCs have been shown to transform into OS by genetic loss of cdkn2 locus [12]. A similar transformation of human MSCs into OS has been seen in the simultaneous knockdown of Rb and overexpression of c-Myc [13].

Although in vitro and in vivo genetic models support the proposition that the unusual differentiation of pre-osteoblasts may be the origin of OS, recent studies suggest that osteogenic progenitors rather than undifferentiated MSCs represent the OS cells of origin [8,14]. The transformation of human osteo-progenitor cells into OS is possible due to the overexpression of the MET gene [15]. Other studies have shown that the osteogenic differentiation stage of bone marrow MSCs (BM-MSCs) inflicts the phenotype of in vivo sarcoma development, implying that BM-MSC-derived osteogenic progenitors might be the cells of origin for OS [16]. Several in vivo genetic model studies support the idea that the abnormal differentiation of osteoblasts is the origin of OS cells. The debate arises from the notion that under physiological conditions, it is challenging to discriminate between MSCs and osteoblasts. While MSCs have the potential to differentiate into osteoblasts, they can transform into OS cells in a specific bone microenvironment [17].

To date, there is no conclusive evidence as to whether OS arises because of the transformation of MSCs, or osteoblastic lineage-committed cells (pre-osteoblasts). The use of advanced gene modification technology and improved gene-editing tools at the cell lineage level may help to explain the origin of OS. Understanding the cellular origin of tumors is key to improving preclinical models for testing and studying their behavior and treatment approaches, which can accelerate translational research efforts to advance outcomes for patients with OS.

### 2.2. MSCs in OS Microenvironment

**Figure 1:**
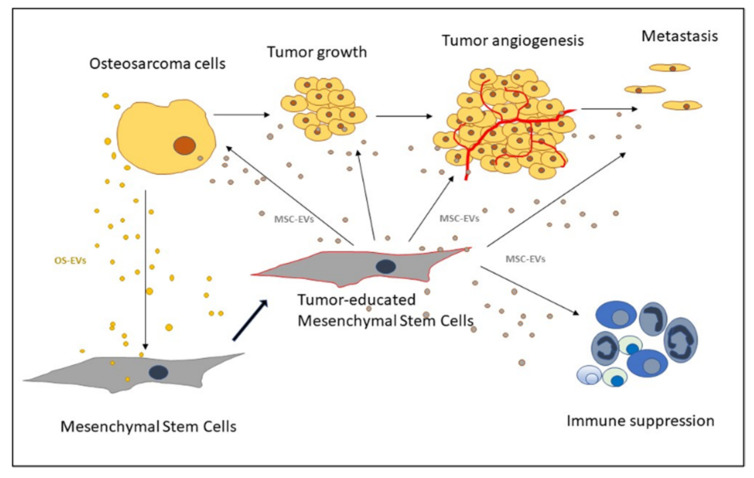
Schematic representation of extracellular vesicles (EVs)-mediated communication between mesenchymal stem cells and osteosarcoma cells (OS) in the OS microenvironment.

OS grows in a bone microenvironment. This is a very specialized, complex, and highly dynamic environment composed of bone cells (osteoclasts, osteoblasts, osteocytes), stromal cells (MSCs, fibroblasts), vascular cells (endothelial cells and pericytes), immune cells (macrophages, lymphocytes), and a mineralized ECM. Crosstalk between OS, bone microenvironment, and ECM involves several signals, induced by multiple cytokines, chemokines, and growth factors carried by EVs. The tumor microenvironment contributes significantly to the development of OS, and MSCs in the TME play an important role in OS growth, progression, metastasis, and drug resistance [18]. Two types of MSCs, from normal tissue and tumor tissue, can accelerate OS growth [19]. Normal-tissue MSCs are recruited to the TME and educated to undergo heterogeneous differentiation into a pro-tumor phenotype (Figure 1). Despite having similar morphological features and differentiation abilities, tumor MSCs derived from OS tissue are more potent effectors of tumor progression compared to normal tissue MSCs.

### 2.3. Recruitment of MSCs to the Tumor Site

OS cells communicate with their microenvironment via paracrine signals that induce the homing of MSCs to the tumor site, where it undergoes changes and produces inflammatory signals that affect the tumor growth, the angiogenesis process, and the immune response (Figure 1). Transforming growth factor (TGF-β) and stromal-derived factor (SDF1) are reported to induce the migration of MSCs towards OS cells, while MCP-1, GRO-α, and TGF-β, in addition to effecting the chemotaxis of MSC, also induce the differentiation of MSCs into cancer-associated fibroblasts, resulting in mesenchymal-to-amoeboid transition [20].

Besides growth factors, proteases derived from ECM and chemokines play a vital role in the recruitment of MSCs at the tumor site. The activation of plasminogen, through the coupling of the Urokinase plasminogen activator and its receptor, induces the migration of MSCs to the tumor site. Moreover, higher plasminogen activity in solid tumors is associated with a greater migration of MSCs, indicating that MSCs recruitment is dependent on plasminogen activity. However, the underlying mechanism through which MSC recruitment is enhanced through plasminogen activation has not been fully explored [21]. MMP-1 is another ECM protease that facilitates MSC recruitment to tumor site [22].

### 2.4. OS Microenvironment-Induced Changes in MSCs

MSCs that migrate to the tumor site or are present in TME change to acquire a phenotype that promotes tumor progression and metastasis (Table 1) [23]. We have shown that OS-derived EVs bring about epigenetic changes and an increased expression of *VEGF-A* in MSCs [24]. OS cells cause the upregulation of IL-6 and VEGF in MSCs, thereby maintaining their stemness, which supports tumor growth and migration [25,26]. Hypoxic conditions in the tumor microenvironment induce tumor cells to switch to anaerobic glycolysis, which results in ECM acidification, which in turn helps in the conversion of normal MSCs into tumor MSCs. Avnet et al. showed that short-term acidosis-exposed MSCs induce NF-κB pathway activation, clonogenicity, and invasion in OS cells in co-culture [27]. Moreover, acidosis was shown to induce a tissue remodeling phenotype of MSC, with an increased expression of colony-promoting factors, chemokines (CCL5, CXCL5, and CXCL1), cytokines (IL6 and IL8), and CXCR4. They further reported that the increased expression of IL6 and IL8 was seen only in normal stromal cells, but not in OS cells, which was similarly confirmed in tumor-associated stromal cells [27]. The modified MSC phenotypes can also account for the development of chemoresistance via IL6 secretion, and this mechanism holds potential for future therapeutic interventions aimed at targeting OS [27].

### 2.5. Transformation of MSCs into Carcinoma-Associated Fibroblasts

Fibroblasts, which are abundant in the stroma of TME, are considered cancer-associated fibroblasts (CAFs). CAFs mediate ECM remodeling during tumor progression and metastasis through proteolysis, crosslinking, and assembly of the ECM, which assists in malignant cell migration and invasion [38].

The conversion of MSCs to CAFs is a complex and multistep biological process involving epithelial-mesenchymal transition. The transformation of MSCs into cancer-associated fibroblasts CAFs is postulated to result from decreased ECM pH and is further dependent on GPR68 and its downstream effectors, G-protein-coupled receptor (GPCR) and YAP. The knockdown of GPR68 or the inhibition of IL-6/STAT3 pathway in MSCs suppress in situ tumor growth and prolong lifespan after cancer grafting [39]. Recently, Lin et al., showed that treating BM-MSC with a conditioned medium from osteosarcoma cell line U2OS transforms MSCs to CAFs via increasing IL-6 expression and the phosphorylation of STAT3, which further promotes the proliferation, migration, and invasion of BM-MSCs [40]. In another in vitro study, Pietrovito et al. showed that BM-MSCs have a strong tropism towards OS cells, with cytokines such as MCP-1, GRO-α, and TGF-β1 being crucial factors in BM-MSC chemotaxis. Upon its recruitment to BME, BM-MSCs differentiates into cancer-associated fibroblasts, resulting in a further increase in MCP-1, GRO-α, IL-6, and IL-8 levels in the tumor microenvironment. These cytokines were shown to promote mesenchymal-to-amoeboid transition (MAT), driven by the activation of the small GTPase RhoA, in OS cells [20].

In addition to IL–6/STAT3′s signaling axis in inducing BM-MSCs to CAFs transition, the Notch and Akt signaling pathway is also reported to play a crucial role in this differentiation. Interestingly, Notch signaling acts upstream of Akt signaling and thus indicates a novel mechanism of BM-MSC-to-CAF differentiation and a potential target for osteosarcoma treatment [41].

### 2.6. MSCs’ Role in Promoting OS Growth and Progression

The supportive role of MSCs in OS growth is backed by many in vivo studies on animal models and by in vitro studies. When injected along with tumor-associated MSCs, OS cells develop larger tumors and more lung metastases, while blocking the paracrine signal of tumor-educated MSCs inhibits OS progression [23]. The expression of Sox2 in MSCs is reported to enhance, while its downregulation inhibits, OS progression [42]. IL-6 and CCL5 secreted by OS-associated MSCs promote OS growth and metastasis [43].

Both MSCs and OS cells reciprocally interact with each other and modulate the BME to favor OS growth (Table 1). OS cells secrete IL–8 and form a signaling circuit, triggering the expression of IL–8 in MSCs, which in turn accelerates the expression of IL–8 in OS cells. The expression levels of both IL-6 and TGF- β are reciprocally dependent upon each other. In particular, TGF-β enhances cell proliferation and ECM deposition and impedes the osteogenic differentiation of MSCs, to retain the cells in an undifferentiated state, which enhances the secretion of pro-tumor cytokines [34,44]. The exposure of osteosarcoma cell lines (Saos-2, MG-63) to condition media from MSCs elevates IL-6 levels, which further activates the STAT3 signaling pathway, resulting in increased tumor growth [25].

### 2.7. MSCs’ Role in Angiogenesis

**Figure 2:**
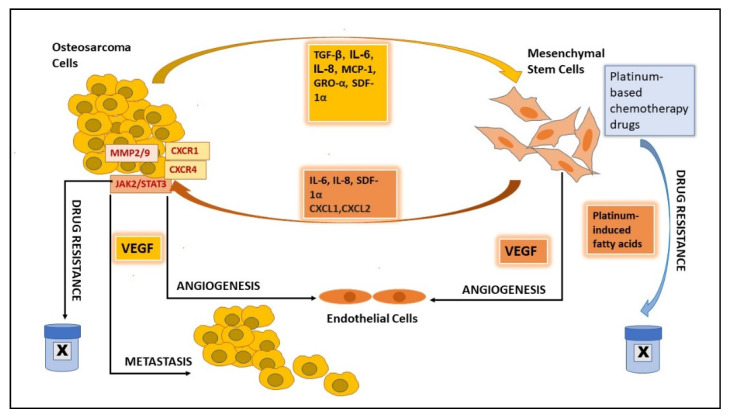
Graphical presentation of the role of mesenchymal stem cells in angiogenesis, metastasis, and drug resistance in osteosarcoma.

The cellular cross-talk between cancer cells and MSCs favors angiogenesis, through which new blood vessels form. This subsequently helps tumor progression by fulfilling the metabolic prerequisites of cancer cells. Growth factors and cytokines, such as TGF-β, MIF, IL-6, and SDF-1, which are produced in the TME and are important in the recruitment of MSCs to TME, further augment tumor growth and metastasis by promoting the neovascularization [19,45] of the tumor (Figure 2). The hypoxia-inducible factor (HIF) signaling pathway activates under hypoxia and tumor cells secrete the angiogenic growth factors for endothelial cell stimulation and migrate the endothelial cells derived from MSCs. These signaling cascades help tumor-associated vascularization, which in turn supports metastasis and tumor progression [46,47]. We observed an increased expression of the angiogenic factor *VEGF* in adipose-derived MSCs (AD-MSCs) when these were treated with EVs derived from OS cells [24].

### 2.8. MSCs’ Role in Metastasis

The presence of tumor-associated MSCs in the microenvironment activates the inflammatory NF-kB signaling cascade, inducing the secretion of cytokine CCL5, which assists in the migration and metastasis of OS in the late stages [47]. Du et al. suggested that MSCs can select for OS cells with high metastatic potential in vivo and claim that CXCR1 is a key target in the regulation of the pulmonary metastasis of OS cells [35]. The metastasis of osteosarcoma cells is reported to be promoted by the upregulation of CXC chemokine receptor 4 (CXCR4) in response to increased VEGF expression in the tumor associated MSCs (Figure 2) [48]. Pelagalli et al. showed that the exposure of OS cells to BM-MSC-conditioned medium elevates the expression level of AQP1 in OS cells and demonstrated that BM-MSCs deployed to the TME promote metastasis and invasion by upregulating AQP1 levels [49].

### 2.9. MSCs’ Role in Drug Resistance

The presence of MSCs in the OS microenvironment is an important part of the OS cell survival mechanism and the development of resistance to anti-cancer drugs [50]. OS induces the expression of IL-6 in MSCs, which in turn activates STAT3 signaling in OS cells and further renders them resistant to the chemotherapeutic drugs, such as doxorubicin or cisplatin, used to treat OS (Figure 2) [25]. Besides, it promotes the survival of OS cells by protecting them from drug-induced apoptosis. The low expression of STAT3 in osteosarcoma patients reduces recurrence after surgery and chemotherapy [25]. Moreover, the inhibition of STAT 3 signaling in mouse models makes OS tumors sensitive to these drugs [51]. Likewise, an association of increased IL-6 levels with the development of chemoresistance has also been observed in several other tumors [52]. Blocking STAT3 activation might thus be a possible solution to overcome drug resistance in OS. MSCs are also reported to induce drug resistance to platinum-based chemotherapeutic drugs systemically. These drugs induce the secretion of platinum-induced polyunsaturated fatty acids (PIFAs), 12-S-hydroxy-5,8,10-heptadecatrienoic acid (12-S-HHT), and 4,7,10,13-hexadecatetraenoic acid (16:4(n-3) in MSCs, leading to drug resistance [53]. The effect can be reversed by COX-1/TXAS inhibitors, such as indomethacin. Clinical trial results from a Phase 1 study using a combination of indomethacin and platinum-based chemotherapy drugs showed a moderate response and were found to be safe [54].

### 2.10. MSCs as Suppressors of OS Growth

MSCs are a double-edged sword for OS; although many studies have established the growth-promoting effects of MSCs in OS, some studies have also proven that MSCs can effectively alleviate and inhibit OS recurrence, proliferation, and metastasis. BM-MSC-derived EVs containing miR-206 are reported to inhibit OS growth by targeting *TRA2B* [55]. The anti-tumor role of MSCs is brought about by the induction of apoptosis, the inhibition of angiogenesis, or the modulation of immune response, which, however, seems to depend upon the MSC source, the tumor site, and the content of the secretome/signaling molecules in the tumor microenvironment (reviewed in [56]).

Interestingly, MSCs suppress tumor recurrence and inhibit the growth of the residual OS cells when injected directly to the primary OS site in mice, whereas, conversely, their intravenous injection promotes lung metastasis [57]. Additionally, the concentration of injected MSCs at the tumor site dictates their effect on tumor growth, with low concentrations of AD-MSCs having an inhibitory effect and higher concentrations having a stimulating effect on tumor growth [58,59]. Some studies have also suggested MSCs as a suppressor of tumor angiogenesis. OS cells (Saos–2 cell line) downregulate the expression of angiogenic factors, such as *CD34, PDGFA, PDGFRA, PDGFRB,* and *VEGF*, in human AD-MSCs when co-cultured, implying that MSCs cannot differentiate in vitro under the induction of tumor cells and do not support tumor angiogenesis in vivo [60].

In another study, Wharton’s jelly-derived MSCs (WJ-MSCs), when cultured with OS cells in a WJ-MSC-conditioned medium, resulted in the inhibition of OS cell proliferation and migration, with the upregulation of pro-apoptotic *BAX* and downregulation of anti-apoptotic *BCL2* and *SURVIVIN*, as well as the upregulation of autophagy genes *ATG5, ATG7,* and *BECLIN1* in MG-63 cells [61].

The tumor-suppressive role of MSCs also seems to depend upon their tissue source. Dental pulp MSCs (DP-MSCs) have more anti-tumor effects compared to BM-MSCs. DP–MSCs exhibit increased osteogenic potential and decreased adipogenic potential, and forms a dentin pulp-like complex that is resistant to tumor transformation. The discrepancies responsible for the lineage commitment and tumorigenesis differences in both cells are attributed to the differences in *PTEN* expression, associated with hypermethylated *PTEN* promoter [62]. The characteristic features of DP-MSCs, including their extremely low tumorigenic potential and unusual cell fate, as well as those of WJ-MSCs, have great potential for the treatment of OS and in regenerative medicine. Investigating the mechanism of the extremely low tumorigenic potential of DP-MSCs could provide insight into their anti-tumor functions and offers a promising future clinical application.

## 3. Extracellular Vesicles as Mediators of MSCs and Osteosarcoma Crosstalk

Extracellular vesicles (EVs) are small lipid-bound vesicles released by cells that carry a heterogeneous cargo, including proteins, RNA, miRNA, other non-coding RNA, DNA, lipids, and metabolites. This cargo can be transferred to other cells and can affect their physiology, thereby playing an important role in cell-to-cell communication. EVs are classified, based on their size, into small-EV (i.e., <200 nm) and medium/large-EV (>200 nm) groups and, based on their origin/biogenesis, they are classified as exosomes (endosome origin), microvesicles (plasma membrane budding) and apoptotic bodies (apoptosis/nuclear origin) [63]. Exosomes fall mainly in the group of small EVs and are the main EVs responsible for intercellular communication.

OS cells can establish crosstalk with resident bone cells by secreting EVs in the bone microenvironment and becoming involved in regulating bone cell proliferation and differentiation, thereby promoting tumor growth [64]. In the TME, the primary tumor cells as well as stromal cells secrete EVs that mediate bidirectional cellular communication [65]. EVs can also be involved in premetastatic niche formation by promoting angiogenesis via the activation of the JAK/STAT signaling pathway [66,67]. The detection of tumor-specific EVs in blood circulation may serve as a diagnostic tumor marker [63].

The development of OS and tumor driving specific genetic alterations are incompletely understood so far. The alterations at the epigenetic level could be the early event occurring in the transformation of MSCs during OS development. Recently, we presented the EV-mediated intercellular crosstalk between MSC and OS, demonstrating that OS-EVs modulate the epigenetic status of MSC, through the hypomethylation of long interspersed nuclear element 1 (*LINE1*), whereas an opposite effect was seen in pre-osteoblasts. Our results indicate that MSCs, but not pre-osteoblasts, are susceptible to OS-EV-mediated epigenetic transformation. Furthermore, OS-derived EVs influenced the AD-MSC’s expression of matrix metallopeptidase 1 (*MMP1*), vascular endothelial growth factor A (*VEGF-A*), and intercellular adhesion molecule 1, which are related to bone microenvironment remodeling [24].

### 3.1. OS-EVs

OS cell-derived exosomes affect the tumor microenvironment by carrying microRNAs, which induce bone remodeling and tumor angiogenesis by promoting osteoclasts differentiation and bone resorption activity. The different proteins, microRNAs, and other non-coding RNAs detected in the EVs cargo derived from OS cells and MSCs that are involved in OS pathogenesis are summarized in Table 2.

The impact of EVs in tumor progression and the metastatic process is exerted through both local and distant intercellular communication. Macklin et al. demonstrated the role of EVs as mediators in the transfer of migratory and invasive characteristics from OS subclones with highly metastatic traits to poorly metastatic cells. This horizontal phenotypic transfer is unidirectional, and the metastatic potential may arise through inter-clonal cooperation. Proteomic analysis of the EVs secreted by highly metastatic OS clonal variants has identified several proteins and G-protein coupled receptor signaling events as potential drivers of OS metastasis and novel therapeutic targets (Table 2) [74].

### 3.2. MSC-EVs

Exosomes derived from BM-MSCs are reported to promote cell proliferation, migration, and the invasion of OS cells by promoting oncogenic autophagy [70]. In a recent study, Li et al. showed that BM-MSC-derived EVs promote the proliferation, invasion, and migration of OS cells via the lncRNA MALAT1/miR-143/NRSN2/Wnt/β-Catenin Axis. BM-MSC EVs carried MALAT1 into OS cells, increased MALAT1 and NRSN2 expressions, decreased miR-143 expression, and activated the Wnt/β-catenin pathway in OS cells. In vivo experiments confirmed that BMSC-EVs promoted tumor growth in nude mice [71].

EVs secreted by MSCs protect OS cells from apoptosis in vitro. Vallabhaneni et al. showed that MSCs under stress (MSC cultured in serum-deprived culture medium) have pro-proliferative, anti-apoptotic, and cell migration effects on OS cells. The microRNAs from the EVs involved in this OS–MSC communication target genes associated with metabolism and metastasis, such as monocarboxylate transporters, bone morphogenic receptor type 2, fibroblast growth factor 7, matrix metalloproteinase-1, and focal adhesion kinase-1. This finding suggests that under conditions resembling nutrient-deficient tumor core tissue, the miRNA content of the EVs released from MSCs are important determinants of a supportive role in osteosarcoma growth [32]. Not surprisingly, microRNAs (miRNAs) are widely related to OS development (reviewed in [77]).

### 3.3. MSC-EVs in Angiogenesis

EVs released by MSCs also affect angiogenesis [78]. MSCs are known to promote angiogenesis under hypoxic conditions, with hypoxia-inducible factor (HIF)-1α contributing significantly to this process. It has been shown that HIF-1α expressing MSCs produce more EVs, can activate Notch signaling, and promote angiogenesis both in vitro and in vivo Matrigel plug studies in mice [79]. Exposing human umbilical vein endothelial cells (HUVECs) to MSC-EVs is shown to induce tube formation in vitro and to mobilize endothelial cells and increase blood flow into Matrigel plug-transplanted in mice. Moreover, MSC-EVs can transfer pro-angiogenic miRNAs, such as miR-30b, from MSCs to endothelial cells and stimulate angiogenesis [80]. On the contrary, in breast cancer cells, MSC-EVs carrying miR-100 are reported to suppress angiogenesis by decreasing the expression of VEGF, while MSC-EVs are reported to enhance VEGF expression in gastric cancer cells [81,82]. However, these contradictory findings may be dependent on the source of MSCs, the EVs’ cargo, and the culture/in vivo conditions. Nevertheless, MSCs secrete EVs that carry miRNAs and proteins and, depending upon the tissue environment conditions, affect angiogenesis.

### 3.4. MSC-EVs in OS Metastasis

In addition to supporting OS growth, MSC-EVs play an important role in OS progression and metastasis. Under specific signals, MSCs are recruited to the tumor site and release factors that help in tumor cell migration and metastasis. In vitro studies have shown that the EVs secreted by MSCs under nutritional stress contain tumor-supportive miRNA, proteins, and metabolites that support cancer cell migration and apoptosis by potentially targeting metastasis-associated genes [32].

Exosomes secreted by AD-MSC promote OS cell growth, invasion, and migration by increasing the expression of COLGALT2 in OS cells in vitro and mouse models [28]. EVs derived from BM-MSC promote OS migration through transferring oncogenic non-coding RNAs and proteins. The transfer of non-coding RNA PVT1 through EVs upregulates ERG expression in OS cells by inhibiting its degradation and ubiquitination and by sponging miR-183-5p in OS cells [72]. BM-MSC-derived exosomal miR-208a promotes OS cell migration and invasion, and exosomal LCP1 promotes OS proliferation and metastasis via the JAK2/STAT3 pathway [73].

The activation of STAT3 in OS by IL-6 is an important mechanism in OS tumor progression and metastasis. EVs secreted by OS cells are shown to express TGF-β on their membrane, which induces IL6 expression in MSCs. The increased IL-6 from MSC promotes tumor growth, STAT3 activation, and lung metastasis from OS [25,29,83]. Another mechanism by which BMSC-EVs promote tumorigenesis and metastasis is the promotion of oncogenic autophagy in OS [70].

Contrary to its supporting role in OS tumor growth and metastasis, BM-MSC-derived EVs can also inhibit OS progression by targeting *TRA2B* via BM-MSC-derived exosomal miR-206. Both in vitro and in vivo results have shown that BM-MSC derived exosomal miR-206 can inhibit the proliferation, migration, and invasion of osteosarcoma cells and induce their apoptosis [55]

### 3.5. MSC-EVs in Immune Response

MSCs are also known to play a vital role in immunomodulation by interacting with immune cells, as well as in modulating the immune response by secreting cytokines and soluble tissue factors [84]. MSC-secreted EVs can affect the activity of immune cells by inducing the expression of anti-inflammatory cytokines while inhibiting that of pro-inflammatory cytokines [85]. A detailed analysis of MSC-EVs proteomes has shown that they include cytokines, chemokines, and chemokine receptors, such as IL10, HGF, LIF, CCL2, VEGFC, and CCL20, that can promote migration of MSC-EVs to injured sites and suppress inflammation [86]. MSC-EV-associated TGF-β and IFN-γ promote the transformation of mononuclear cells into Treg cells [87]. MSC-EVs affect the proliferation of immune cells, increase the levels of CXCL8 and MZB1 RNA, and decrease IgM in B-cells [88].

Tumor-associated macrophages (TAM) are the most common type of immune cells infiltrating OS tumors and play an important role in OS immunology. Depending upon environmental signals, macrophages can change from a tumor-suppressive M1 state to a more tumor-promoting and immune-suppressive M2 state [89]. Conditioned media from M2 macrophages increase osteoblast differentiation from MSCs [90].

EVs secreted by MSCs cultured in serum-deprived mediums and low-oxygen conditions are enriched in metabolites that have immunomodulatory effects, M2 macrophage polarization, and induction of T lymphocytes [91]. MSCs treated with conditioned media from M1 macrophages were found to promote tumor growth in a mouse model by converting tumor-associated macrophages into immunosuppressive M2 type by IL-6 secretion [92]. Moreover, M2 macrophages have been shown to increase the cancer stem cell characteristics of OS in vitro and targeting M2 tumor-associated macrophages could be useful in OS therapy [93]. The potential of the TAM targeted approach for OS treatment is increasingly accepted. Exosomes from M1 macrophages engineered to target IL-4 receptors of TAM have been shown to transform TAMs into M1 phenotypes and suppress OS growth in vivo by increasing immune response to the tumor [94].

## 4. MSCs in OS Therapy

MSCs from different tissue sources and engineered MSCs are being explored for their application in OS treatment. The unique ability of MSC to engraft and home in on tumor stroma makes them attractive targeted delivery vehicles to carry therapeutic drugs to the tumor stroma. TNF-related apoptosis-inducing ligand (TRAIL) delivered by AD-MSCs have an anti-tumorigenic effect on osteosarcoma. AD-MSC-delivered TRAIL efficiently kills OS cells, since MSCs confer a longer half-life with stable TRAIL delivery and the secretion of synergizing factors [95,96]. A clinical trial (NCT03298763) is underway to access the efficacy of stem-cell-expressing TRAIL (MSCTRAIL) combined with chemotherapy in metastatic non-small cell lung cancer patients. Similar clinical studies could also be possible in OS.

MSCs transduced with adenoviruses harboring the osteoprotegerin (OPG) gene, when injected into mice through the tail vein, have been shown to migrate to the tumor site and produce OPG locally, thereby inhibiting OS growth. Moreover, MSC-OPG administration is reported to cause no increase in serum OPG levels, indicating fewer side effects of systemic administration, and reveals the tumor suppressor role of MSC-OPG [97]. Similarly, MSCs transduced with an adenoviral vector carrying the Interleukin-12 (IL-12) gene injected into mice bearing Ewing’s sarcoma have been shown to localize and produce IL-12 selectively at the tumor site, inhibiting tumor growth. Moreover, these MSCs also migrated to the lungs, spleen, and liver without harming these organs [98]. Combining the anti-tumoral effect of IL-12 and the ability of MSCs to migrate to the tumor site can be an efficient way to deliver IL-12 locally to tumors.

MSCs can also be used to deliver anti-tumor drugs. MSCs uploaded with nanoparticles and photosensitizers have been shown to trigger OS cell death in vitro upon specific photoactivation via the release of reactive oxygen species (ROS). However, antineoplastic agents, which are used in MSC delivery systems, may kill MSCs, leading to the failure of this technique [99].

MSCs also have applications in photodynamic therapy. MSCs loaded with nanoparticles (AlPcS4@FNPs) and their photoactivation have shown encouraging results in suppressing tumor growth in vitro and in vivo murine models for OS treatment. Photodynamic therapy is especially suited to patients with chemoresistant tumors or unresectable small tumors [100].

While MSCs are promising vectors for antineoplastic agent delivery and bone defect regeneration after bone sarcoma resection, there are also some concerns regarding their side effects. They can accelerate the proliferation of cancer cells, induce pulmonary metastases, and increase chemoresistance to doxorubicin and cisplatin when interacting with cancer cells in TME. However, the use of transduced MSCs to express specific anti-cancer molecules, such as TRAIL, OPG, IL-12, or the CD/5-FC prodrug selectively to the tumor site, suggest that MSCs could be suitable for delivering drugs in OS. However, more research is required to expand the engraftment efficiency of MSCs in tumors. The use of MSCs carrying oncolytic adenovirus is being tested to treat childhood tumors, including different sarcomas, and the first human clinical trial carried out using autologous bone marrow-derived MSCs carrying oncolytic virus has shown good results [101]. Preclinical testing using the same strategy, employing MSCs carrying oncolytic virus together with granulocyte-colony stimulating factor, have also shown promising results in osteosarcoma, demonstrating increased immune infiltration and reduction in tumor growth [102].

### MSC-EVs in OS Therapy

EVs are known for transporting miRNAs to other cells and subsequently affecting their physiology. They are therefore being tested as a vehicle to deliver tumor-suppressive miRNA to target cancer cells. Zhang et al. revealed that AD-MSC-derived miR-101 can suppress OS via downregulation of *BCL6* and proposed that AD-MSC-derived miR-101-enriched EVs could represent a potential innovative therapy for metastatic OS [75].

Similarly, MSCs-derived exosomes carrying miR-150 suppresses the proliferation and migration of osteosarcoma cells by targeting *IGF2BP1*. Further validation of the anti-tumor effect of MSC-Exo-miR-150 in vivo indicates that it could serve as a potential therapeutic agent for OS patients. However, the efficiency and safety of MSC-Exo-miR-150 in the treatment of OS remains to be studied [103].

Another approach for miRNA delivery via MSCs for OS therapeutics is the introduction of synthetic miR-143 into MSCs, which increases the secretion of exosomal miR-143 in the conditioned medium. Exosome-formed miR-143 is easily transferred into recipient cells and suppresses the migration of OS cells [76]. Although the therapeutic application of exosome-formed miRNAs requires further in vivo studies, exosome-formed miRNAs offer great potential for the targeted delivery of miRNAs in miRNA-supplementation therapies for various diseases, including cancers such as osteosarcoma.

Exosome-based cancer therapies, especially those related to cancer vaccines and immunotherapies, have been tested in clinical trials [104]. A clinical trial of immunotherapy combining metronomic cyclophosphamide (mCTX) treatment and tumor antigen-loaded dendritic cell-derived exosomes (Dex) for treating non-small-cell lung cancer reached phase 2 (NCT01159288). Exosome-based cancer therapies involving engineered MSCs’ exosomes are already undergoing clinical trials. A phase 1 clinical trial (NCT03608631) for treating metastatic pancreatic cancer patients with KRAS G12D mutation with MSC-derived exosomes containing KRAS G12D siRNA (iExosomes) is ongoing.

## 5. Conclusions

MSCs make a significant contribution to the development and progression of OS. MSCs and OS cells reciprocally communicate with each other via paracrine signaling, mediated by cytokines, growth factors, chemokines, and EVs. This communication induces MSC migration and its transformation into a tumor-associated phenotype that promotes angiogenesis and metastasis, and confers drug resistance. Studies on the effects of MSCs on tumor growth have produced conflicting conclusions. However, the tissue origin of MSCs, and the interaction between MSCs, OS cells, and the tumor microenvironment, seems to dictate the pro-tumorigenic or tumor-inhibiting pathways of MSC in OS. EVs aid in intercellular communication via the transport of miRNAs and proteins, and a deeper investigation into this interaction will greatly help in finding new drug targets and therapies for OS. The ability of MSCs to home in on and graft tumors provides a promising opportunity to deliver tumor-suppressive miRNAs or other noncoding RNAs, proteins, or drugs via MSC-EVs or through viral transduction. Further studies on safety, MSC tissue source, and delivery modes are needed to test the possible utility of MSC-EV-based OS therapies.

## Figures and Tables

**Table 1:** Interaction of mesenchymal stem cells (MSCs) and osteosarcoma cells (OS).

OS Signal	Effect on MSCs	MSC Signal	Effect on OS	Reference
		Exosomes	Increase in COLGALT2 and proliferation.	Wang et al. 2020 [28]
		CM ^1^/co-culture	Increase in MMP2/9; STAT3 activation. Increased proliferation, invasion, and metastasis.	Wang et al. 2017 [29]
IL-8 (co-culture)	Increased IL-8.	IL-8 (co-culture)	Increased IL-8. Increased metastatic potential.	Kawano et al. 2018 [30]
		IL-6 CM/co-culture	Increase in MMP2/9; JAK2/STAT3 activation. Increased proliferation, migration, and doxorubicin resistance.	Lu et al. 2021 [31]
MCP-1, GRO-α, and TGFβ	Mesenchymal-to-amoeboid transition. Increase in MCP-1, GRO-α, IL-6, and IL-8 in the tumor environment.		Increased migration, invasion, and trans-endothelial migration.	Pietrovito et al. 2018 [20]
OS-EVs	LINE-1 hypomethylation increased VEGF-A.			Mannerström et al. 2019 [24]
		CM	STAT3 activation. Promote survival and drug resistance.	Tu et al. 2016 [25]
		MSC-EVs (under stress)	Increased migration. Apoptosis resistance.	Vallabhaneni et al. 2016 [32]
Co-culture	Increased TGFβ.	Co-culture IL-6	Increased OS proliferation, stemness & migration.	Cortini et al. 2016 [26]
		IL-6	STAT3 activation. Increased proliferation and metastasis.	Tu et al. 2012 [33]
CM/TGFβ	Increased IL-6, VEGF. Inhibit osteogenic differentiation.			Tu et al. 2014 [34]
		IL-8	CXCR1/Akt activation. Promotes metastasis.	Du et al. 2018 [35]
EVs/TGFβ	Increased IL-6.	Tumor-educated MSC	Activation of STAT3 signaling.	Baglio et al. 2017 [23]
		EVs	Cell growth under hypoxia. Activation of PI3K/AKT & HIF-1α.	Lin et al. 2019 [36]
		EVs	Activation of Hedgehog signaling. Tumor growth.	Qi et al. 2017 [37]

^1^ CD, conditioned medium.

**Table 2:** Role of microRNA, non-coding RNA, and proteins associated with osteosarcoma (OS) and mesenchymal stem cell (MSC)-derived extracellular vesicles (EVs) in OS pathogenesis.

Source of EVs	miRNAs/other RNAs in EVs	Proteins in EVs	Function	Reference
OS cells	miR-146a-5p, miR-10b-5p, miR-143-3p, miR-382-5p, miR-150-5p, miR-125b-5p, miR-27a-3p, miR-145-5p, miR-26a-5p, miR-93-5p, miR-21-5p, miR-92a-3p, and miR-106a-5p	serpin-E1, serpin-F1, TIMP-1, thrombospondin-1, urokinase-type plasminogen activator (uPA), VEGF, pentraxin-3, PDGF-AA, angiopoietin-2, coagulation factor-III, CD26, CD105, endostatin, endothelin-1, and HB-EGF	Angiogenesis.	Perut et al. 2019 [68]
OS cells & tissue	lncRNA OIP5-AS1		Angiogenesis.	Li et al. 2021 [69]
OS cells	miR-148a-3p and miR-21-5p		TME remodeling.	Raimondi et al. 2020 [64]
OS cells		TGFβ	Increase IL6 in AD-MSCs, tumor growth, STAT3 activation, and lung metastasis. Autophagy.	Baglio et al. 2017, Tu et al. 2012, Huang et al. 2020 [23,33,70]
BM-MSC	lncRNA MALAT1		Proliferation, invasion, and migration of OS cells via lncRNA MALAT1/miR-143/NRSN2/Wnt/β-Catenin Axis.	Li et al. 2021 [71]
BM-MSC	non-coding RNA PVT1		OS migration by upregulating ERG and sponging miR-183-5p in OS cells.	Zhao et al. 2019 [72]
BM-MSC	miR-206		Tumor suppression and apoptosis.	Zhang et al. 2020 [55]
BM-MSC	microRNA-208a LCP1		OS cell migration & invasion. OS proliferation and metastasis via the JAK2/STAT3 pathway.	Qin et al. 2020 [73]
Highly metastatic OS cells		NPM1, CCT2, CCT4, CCT6A, CCT8, VIM, CLTC, COL6A2, HNRNPC, PKM, ACTN4, MYH10, PAICS, VCP, ANXA1, ACLY	Metastasis.	Macklin et al. 2016 [74]
Engineered AD-MSC	miR-101; miR-150 synthetic miR-143		OS Therapy. Suppress OS growth.	Zhang et al. 2020; Shimbo et al. 2014; Xu et al. 2020; [75,76]
